# Effect of *Myrtus communis* L. Plant Extract as a Milk Supplement on the Performance, Selected Blood Parameters and Immune Response of Holstein Calves

**DOI:** 10.3390/ani14050725

**Published:** 2024-02-26

**Authors:** Cangir Uyarlar, Abdur Rahman, Umit Ozcinar, İbrahim Sadi Cetingul, Eyup Eren Gultepe, Ismail Bayram

**Affiliations:** 1 Department of Animal Nutrition and Nutritional Diseases, Faculty of Veterinary Medicine, Afyon Kocatepe University, 03204 Afyonkarahisar, Turkey; cangiruyarlar@hotmail.com (C.U.); umitozcinar@gmail.com (U.O.); sadicet@yahoo.com (İ.S.C.); eegultepe@gmail.com (E.E.G.); ibayram@aku.edu.tr (I.B.); 2 Department of Animal Sciences, University of Veterinary and Animal Sciences, Jhang Campus, Lahore 54000, Pakistan

**Keywords:** calf, myrtus, performance, reproductivity, blood

## Abstract

**Simple Summary:**

The growing female calves are the future of the dairy farms and their balanced growth yields benefits in their adult lives. Natural herbal products and their extracts are considered as digestive stimulants, immune enhancers and anti-oxidants. The additions of these phytogenic extracts have proven to be beneficial in animals. This study involved the addition of myrtus leaves and root extract in the milk of the suckling calves and observed its benefits on their health, performance and immune status in the following days. It is concluded that the addition of the myrtus extract results in the improvement in the overall performance, reproduction and health status of the animals.

**Abstract:**

This research aimed to understand the effects of adding myrtle plant extract obtained from its leaves (MPEL) and roots (MPER) to the milk fed to suckling female Holstein calves, focusing on performance, reproduction, selected blood parameters and immune response. The 50 Holstein female calves, one week of age, were divided into five groups: one group received no plant extract (Control), while the others were supplemented with myrtle plant extracts at doses of 25 mL/day leaf extract (MPEL-25), 25 mL/day root extract (MPER-25), 50 mL/day leaf extract (MPEL-50) and 50 mL/day root extract (MPER-50) for each calf in each treatment group. The extracts were given along with the milk to the experimental groups for 60 days, and for an additional 12 days post-weaning. The results reveal that the feed consumption and live weights increased significantly. Significantly higher leukocyte counts were observed in the 50 mL/head × day myrtle groups, and a higher IgG concentration was also noted in the MPER-50 group compared to the other groups. The serum non-esterified fatty acid (NEFA) concentration significantly decreased in the MPEL-50 and MPER-50 groups, whereas the betahyrdoxy butyric acid (BHBA) concentration increased and the serum glucose concentration significantly decreased with myrtle supplementation. In conclusion, it was determined that the performance, immune system and negative energy balance compensation of female Holstein calves were positively affected by administering extracts obtained from the leaves and roots of the *Myrtus communis* L. plant at dose levels of 25 and 50 mL/head × day for 72 days, without causing any side effects.

## 1. Introduction

A successful calf production and feeding program significantly influences the growth and health of dairy calves, impacting their future performance. Early life management and feeding practices, referred to as perinatal programming, exert three to seven times greater influence on future milk production than maternal choices. Implementing rational nutrition and management practices in heifers before puberty increases their future performance [1]. In dairy farming, the colostrum and milk-feeding period for newborn calves is a critical phase during which they are highly susceptible to nutritional issues and pathogens. Antibiotics are employed to address such challenges, aiming to enhance the performance of calves slated for future breeding or fattening. This approach also seeks to reduce drug costs and achieve economic profitability. However, as in monogastric animals, administering antibiotics to calves for these purposes results in the development of resistance against pathogenic microorganisms, primarily due to the intensive and widespread use of antibiotics. Research indicates that pathogenic microorganisms acquire antibiotic resistance when antibiotics are administered at doses lower than therapeutic levels [2].

For these reasons, many feed additives, such as herbal extracts, probiotics, prebiotics, and synbiotics, are used as alternatives to antibiotics in both milk and animal feed [3,4]. Common Myrtus (*Myrtus communis* L.) is a flowering plant species belonging to the Myrtaceae family. This evergreen shrub is native to Southern Europe, North Africa, West Asia and the Indian subcontinent, and it can also be cultivated [5]. The myrtle plant has been utilized for centuries in the treatment of various diseases [6]. Traditionally, it has been employed to address conditions such as diarrhea, peptic ulcers, hemorrhoids, inflammation, wounds, skin issues and respiratory diseases [7]. Clinical and experimental studies have demonstrated its antioxidative, anticancer, antioxidant, antibacterial, antifungal, hepatoprotective, anti-diabetic, antiviral properties, as well as neuroprotective activity [8,9]. In recent years, the oils and extracts of the myrtle plant have been utilized to enhance performance in broilers [10,11], laying hens [12], rabbits [13] and rats [14,15]. Additionally, it has been employed as a chemotherapeutic agent in quails [16]. The medicinal attributes of myrtle are linked to its chemical components, which include tannins, alkaloids, flavonoids, and essential oils [17]. The fruits primarily contain organic acids, essential oils, tannins, sugars, flavonoids, citric acids and malic acids. Furthermore, the leaves contain flavonoids and essential oils, such as tannins, quercetin, catechin and myricetin derivatives [18]. Ozek et al. [19] reported that the most crucial components of myrtle essential oil include myrtenol, myrtenol acetate, limonene, linalool, α-pinene, 1,8-cineole, β-caryophyllene, p-cymene, geraniol, nerol, phenylpropanoid and methyl eugenol. Hayder et al. [20] demonstrated that myricetin-3-o-galactoside and myricetin-3-o-rhamnoside, isolated from the leaves of Myrtus Communis, can inhibit xanthine oxidase activity, lipid peroxidation and scavenge free radicals. 

Given the reasons outlined above, this study was conducted to investigate the effects of extracts obtained from the leaves and roots of the myrtle plant on the performance, fertility, blood metabolites and immune response in Holstein calves.

## 2. Materials and Methods

This study was conducted on newborn calves to weaning. In the following, we put forward our research trial preparation.

This study received approval from the Animal Care and Use Committee of Afyon Kocatepe University, Turkey (No. 49533702/40). The research was conducted at a dairy farm in the Çanakkale province, located at 26°24′ E Longitude and 40°08′ N Latitude, where approximately 1000 calf births occur annually. This study involved 50 female Holstein calves that were newborns. Within two hours of birth, each calf was provided with at least 2 L of colostrum, milked from their mothers at 38 °C and verified with a colostrometer. Subsequently, on the first day, the same quantity of colostrum was continuously provided at 8 h intervals. On the second and third days, each calf received colostrum milked from its mother, equivalent to 10% of its birth weight, in two meals in the morning and evening (8:30, 20:30). After the colostrum period, milk feeding commenced. At seven days of age, the calves were weighed, and blood samples were collected for the study. 

Diet and Design of the Study:

Throughout the study, all calves were individually housed in hutches, with barley straw used as bedding material, cleaned weekly. They were provided calf starter feed and water ad libitum through individual feeders and water drinkers. Calf starter feed was formulated according to the guidelines of NRC [21] and feed samples were analyzed by Weende analysis (dry matter, crude ash, crude protein, crude fiber, crude fat) according to the methods described in AOAC [22] and acid detergent fiber (ADF) and neutral detergent fiber (NDF) analysis were also performed by the Van Soest method [23]. Metabolizable energy was also determined by the NRC method [21]. The ingredient and chemical composition of calf starter feed is detailed in Table 1. Feeders were replaced daily, and leftover feed was weighed and recorded. The animal experiment phase of the study extended for 72 days, representing the milk-drinking period of the calves. During the first 60 days, all calves received 5 L of milk produced on the farm, divided equally between morning and evening (8:30, 20:30). From days 60 to 66, the weaning process commenced, with two meals per animal (1.5 L each) provided, and a single meal per animal (1.5 L) from days 66 onward. Seven-day-old calves were divided into five groups, with each group containing 10 calves. The formation of the following groups was based on the dosage of the extract (Biyoderm^®^, ArsArthro Biotechnologies Inc., Ankara, Turkey) derived from the leaves (MPEL) and roots (MPER) of the myrtle plant during the 72-day milk-drinking period.

The following dietary design was applied:

Group 1: Control—No additional application was administered.

Group 2: Received 25 mL/head × day of the extract (Biyoderm^®^) from myrtle plant leaves (MPEL-25).

Group 3: Received 25 mL/head × day of the extract (Biyoderm^®^) obtained from the myrtle plant roots (MPER-25).

Group 4: Received 50 mL/head × day of the extract (Biyoderm^®^) obtained from myrtle plant leaves (MPEL-50).

Group 5: Received 50 mL/head × day of the extract (Biyoderm^®^) obtained from myrtle plant roots (MPER-50).

Myrtle plant extract was given daily to each group by mixing in milk, in specified quantities using a milk bottle feeder up to the 60th day of the experiment. Between the 60th and the 72nd day, when the weaning period commenced, myrtle extract was provided to the experimental groups in precise amounts via a feeding bottle separately.

Myrtle plant’s extract (Biyoderm^®^) Solution Preparation and analysis:

Myrtle extract was procured from a commercial company (Biyoderm^®^, ArsArthro Biotechnologies Inc., Ankara, Turkey). The brief process of extract preparation is outlined here. Two grams of dried myrtle plant (*Myrtus communis* L.) leaf or root parts is initially shredded, using a mechanical shredder. The shredded parts are then immersed in sterile distilled water (01 part myrtle and 99 parts water) with a pH of 4.5 at 98 °C for 22 min. Afterward, the solution is allowed to cool, and its acidity with pH 4.5 is balanced using Na_2_HPO_4_ as a buffer solution, if needed. The resulting solution is filtered with medium flow, ash content < 0.01% and a thickness of 0.2 mm. Subsequently, it is bottled and stored in the refrigerator until needed. Liquid chromatography (LC-MS/QTOF-Agilent Technologies, Santa Clara, CA, USA) and a mass spectrometer analysis instrument (Orbitrap-Thermo Electron, Bremen, Germany) were used to determine active ingredient composition. Moreover, Agilent Poroshell 120 ECC183.0 × 100 mm, 2.7 μm colon were also used by 10 Mm ammonium formate 0.1%, formic acid and water with MFB 0.1% formic acid MeOH (Gas Temperature 325 °C, Gas Flow 10 L/min, Nebuliser 45 psig) for active ingredients determination (Agilent Technologies, Santa Clara, CA, USA). All the ingredients were reported to have negative and positive polarization with both instruments. Amino acid analyses were conducted using an HPLC device (Shimadzu, Columbia, MD, USA) and mineral analyses were conducted on the Shimadzu ICPMS-2030 system (Shimadzu, Columbia, MD, USA), equipped with a mini torch for low Ar gas consumption. The composition of the extract is given in Table 2.

Health monitoring and vaccination of animals:

The health of all calves was monitored by a veterinarian at least once a day, and immediate treatment was provided to calves exhibiting symptoms of diarrhea and respiratory diseases. For monitoring symptoms, the scoring criteria outlined by Heinrichs et al. [24] were followed. Throughout their treatment, the milk and feed consumption of sick animals continued to be monitored, documenting the duration of treatment and the total recovery days. When the calves reached 21 days of age, all of them received a subcutaneous administration of a 5 mL Bovilis (Bovipast, MSD Animal Health, Walton, UK) vaccine in the neck area to protect them against respiratory system diseases. A vaccine booster was administered in the same manner 21 days after the initial application, when the calves were 42 days old. This vaccine contains inactive BRS virus (EV 908 strain) with a value of 105.5 TCID50, inactive Parainfluenza 3 virus (SF-4 Reisinger strain) with a value of 105.5 TCID50 and 9X109 inactive Pasteurella (Mannheimia) hemolytic cells (A1 serotype).

Blood samples collection:

Two blood samples were collected from all calves using vacuum tubes from the jugular vein, one containing EDTA and the other containing no EDTA, on the 7th, 21st, 42nd, 60th and 72nd day of life. Complete blood analysis (including red blood cell count, RBC; hematocrit, HCT; hemoglobin, Hb; mean corpuscular volume, MCV; mean corpuscular hemoglobin, MCH; mean corpuscular hemoglobin concentration, MCHC; platelet, PLT, PDW; platelet distribution width and white blood cells, WBC; Neutrophil, Lymphocyte, Monocyte, Eosinophile and Basophile) were promptly conducted following blood collection, utilizing kits named Mindray BC 2800Vet brand device (Mindray Medical International Ltd., Shenzhen, China). Blood samples, drawn into empty tubes, underwent centrifugation at 4200× *g* for 10 min in the laboratory, facilitating the extraction of serum samples. These serum samples were then frozen at −20 °C for subsequent relevant analysis. Upon the completion of the animal testing phase of the study, the frozen serum samples were thawed at room temperature. Immunoglobulin G (IgG), Glucose, non-esterified fatty acid (NEFA), beta hydroxy butyric acid (BHBA), total protein and blood urea nitrogen (BUN) analyses were carried out using the corresponding kits (Glucose: Roche, France, Cat. No: 04404483190; Triglycerides: Roche, France, Cat. No: 20767107322; Cholesterol: Roche, France, Cat. No: 03039773190; BUN: Roche, France, Cat. No: 04460715190; Total Protein: Roche, France, Cat. No: 03183734190; IgG: Bioassay Technology Laboratory, Cat. No: E0010Bo; NEFA: Randox, UK, Cat. No: FA115; BHBA: Randox, UK, Cat. No: RB1008) on the Chemwell (2910) automatic analyzer (Awareness Technology, Palm City, FL, USA). All calves were weighed on the day of blood collection, and daily records were maintained for calf starter feed consumption. Weende analysis, metabolizable energy and Van Soest analysis of feed were performed [21,22,23] and for the milk used throughout the study, daily analyses were conducted for dry matter, fat percentage, protein percentage and lactose percentage using MilkoScan FT 120 (Foss Analytical, Hillerod, Denmark).

Phase 2 of study after weaning:

After the trial phase of the study, all calves were socialized by being placed in the same paddocks. They continued to stay in this paddock until the day of conception, consuming rations ad libitum, as indicated in Table 3. Heifer feed was formulated according to the guidelines of NRC [21] and feed samples were analyzed by Weende analysis (dry matter, crude ash, crude protein, crude fiber, crude fat), while metabolizable energy and Van Soest (ADF and NDF) analysis were also performed by the methods described in the literature [21,22,23]. Daily health checks were conducted until they became pregnant, and immediate treatment was provided to animals displaying signs of disease. At 120 days old, all animals received the conjunctival Brucella abortus (S-19) vaccine, followed by the foot-and-mouth vaccine at 160 days old, and the Hiprabovis 4 vaccine (a mixed vaccine against IBR, BVD, PI3, and BRS agents, Hipra, Spain) at 190 days old. Weights were recorded at the ages of 300 and 420 days, and their development was monitored. Starting from the age of 330 days, a veterinarian conducted gynecological examinations, including the assessment of ovarian and follicle development and the determination of the first heat, through rectal examination. Artificial insemination was transrectally performed on animals in heat. Estrus monitoring was based on natural signs such as jumping on other animals, stopping when jumped on, chara flow, etc. To ensure uniformity in insemination success, commercial semen named Garza (Atafen, Turkey) was used for all animals, and inseminations were consistently performed by the same veterinarian. Pregnancy examinations were conducted transrectally using ultrasonography twice, on the 28th and 45th days following insemination. In animals found to be pregnant at the first examination, the second examination confirmed the continuation of pregnancy. Records were kept for age at first insemination, age at conception and the number of inseminations per conception.

**Data Analysis:** The body weight (7–72nd day), feed intake, FCR, biochemical and hematological results were assessed using PROC MIXED of SAS (SAS Institute, Cary, NC, USA) as repeated measures. The treatment, day and their two-way interactions were considered fixed effects in the model, with the random effect being the calf. Due to unequal sampling time intervals, spatial power was applied in the covariance structure of the model. Degrees of freedom were calculated using a between–within model [25]. Similarly, the evaluation of BW on day 300, BW on day 410, the day of the first AI and the number of AIs per conception was conducted using PROC MIXED of SAS, with the treatment as a fixed effect and the calf as a random effect. Normal distributions were examined with the Shapiro–Wilk test using PROC UNIVARIATE SAS (SAS Institute, Cary, NC, USA). For data with non-normal distribution, log-transformation was performed before analysis. Studentized residuals were determined for all fixed effects and interactions, and outliers (studentized residue < −4 or > 4) were removed from the model. Multiple comparisons were carried out using the PDIFF order of SAS.

Contrasts between control and treatment diets (0% vs. 25% + 50+) were evaluated for linear and quadratic trends, considering either leaf or root extracts. Coefficients of contrasts were calculated using PROC IML of SAS [26] and CONTRAST statement of PROC MIXED SAS (SAS Institute, Cary, NC, USA) was used within the similar model. All data were presented as least square means in the tables. The significance level was set at *p* ≤ 0.05 for main effects and at *p* < 0.15 for trend analysis.

## 3. Results and Discussion

When evaluating the differences between groups in terms of live weight and feed conversion ratio, which are performance parameters, no significant increase was observed during days 7–72; however, numerically an increase in weight was observed in the trial groups on the 72nd and 400th day (Table 4). Significant positive differences were noted in body weight on the 300th day in the MPER-25, MPEL-50 and MPER-50 groups as compared to the control. By giving myrtle plant extracts at dose levels of 25 and 50 mL/head × day along with drinking milk, feed consumption increased in the experimental groups (*p* = 0.002), and the feed conversion ratio did not differ as compared to the control group. There was no effect on the artificial insemination and conception rate (Table 4). Previous studies have reported similar positive effects, such as increased feed consumption and egg production in laying hens when myrtle plant extract was added to drinking water at different concentrations [12]. Consistent with these findings, Bardzardi et al. [10] observed increased feed consumption and live weight in broilers when myrtle extract was added to the rations. Similarly, when different concentration levels of myrtle oil were added to broiler rations, feed consumption increased in the 2000 mg/kg and 5000 mg/kg extract groups, while a decrease in live weight was observed [16]. Bülbül et al. [27] reported increased egg production in quails with myrtle plant extract at the dose level of 1000 mg/kg. In contrast, Saei et al. [28] found that myrtle plant extract did not significantly change live weight gain in broilers compared to the control group, but it reduced feed consumption and improved the feed conversion ratio. Taee et al. [29] reported higher live weight gain and improved feed conversion ratio in rainbow trout by adding 1% myrtle powder to the rations. The positive effects on growth and feed consumption in calves in the present research may be attributed to the antioxidant, antibacterial, antifungal and anti-inflammatory properties of myrtle plant extract. In a study [30], myrtle plant leaves were found to have a total amino acid content of 11.65%, with glutamic (1.56%) and aspartic acids (1.27%) being highlighted as dominant among the amino acids. The higher feed consumption in the MPEL-50 and MPER-50 groups suggests that this increase in feed intake may be due to glutamic acid, which is the predominant amino acid in the myrtle plant. Consequently, due to the high concentration of glutamic acid, an appetizing substance in calves, the appetite center may be stimulated, leading to increased live weight through greater feed consumption. It is well documented that certain herbs and spices are very rich in glutamic acid content [31]. However, glutamic acid acts as umami substance and enriches the taste of food by activating the detection components of taste buds [32]. According to Camilleri [33], umami is one of the five basic tastes and it stimulates taste receptors and enhances the apatite. Glutamic acid is known to act as an excitatory neurotransmitter and is also used as a flavor enhancer, such as monosodium glutamate (MSG), which is considered to provide a fifth taste in Japan (a distinct savory taste of deliciousness). 

The myrtle plant extract was numerically found to shorten the first insemination time and reduce the number of inseminations (Table 4). Although statistically insignificant, when the trial group calves were utilized as breeding heifers, their time of conception occurred earlier, resulting in an earlier birth time, lactation and peak period. The improved fertility parameters in the experimental groups are likely attributed to the active components with estrogenic antioxidant properties in the myrtle plant extract. Estrogens, the primary female hormones, play a crucial role in both menstrual and estrous reproductive cycles. Oral administration of myricetin at a dosage of 100 mg/day induced estrogenic activity, as evidenced by an increase in uterine weight and length in immature Wistar albino rats, when compared to controls (ethinyl estradiol, ethinyl estradiol + tamoxifen and genistein) [34]. The active components present in the myrtle plant are reported to have a positive effect on fertility, not only in females but also in male animals [35]. Abidli et al. [35] reported that myrtle extract could contribute to preventing reproductive disorders in male mice due to the phenolic compounds it contains. Furthermore, it was noted that myrtle leaf powder improved reproductive performance, seminal plasma TAC and blood SOD activity in Arabian rams, with the most favorable outcome observed in the group given myrtle leaf powder at 25 g/kg [36]. 

**Figure 1:**
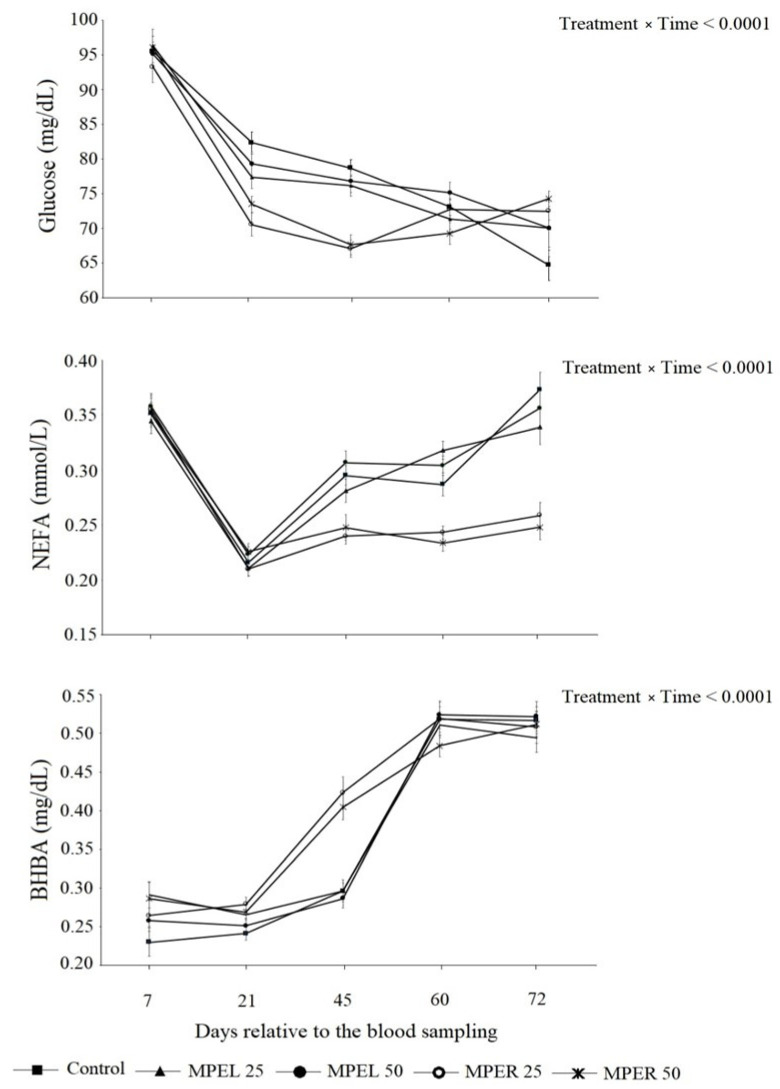
Effect of myrtle plant extract supplementation on serum glucose, NEFA and BHBA in calves.

Significant differences were observed between the groups in terms of glucose (*p* = 0.01), BHBA (*p* = 0.0004), NEFA (*p* < 0.0001) and IgG (*p* = 0.05) values. However, no significant difference was observed on total protein (TP) and blood urea nitrogen (BUN) values (Table 5). As expected, blood glucose concentration levels decreased in all groups over time. According to colostrum and intense milk feeding in the first days of life, the blood glucose concentration level remained high for a few weeks. Therefore, glucose values started to decrease due to the slower digestion of solid feeds than milk as feed intake increased after 3 weeks of life. This phenomenon was achieved in all groups. However, blood glucose values were lower in MPER25 and MPER50 than in other groups for the 21st and 42nd days of life (*p* < 0.0001). On the other hand, it remained similar for all groups on other sampling days (60th, 72nd) (Figure 1). According to colostrum and intense milk feeding in the first days of life, blood glucose concentration levels remained high for a few weeks. Therefore, glucose values started to decrease due to the slower digestion of solid feeds compared to milk as feed intake which increased after 3 weeks of life. This phenomenon was achieved in all groups. However, blood glucose values were lower in MPER25 and MPER50 than in other groups for the 21st and 42nd days of life (*p* < 0.0001). Moreover, they remained similar for all groups on other sampling days (60th, 72nd) (Figure 1). Blood NEFA concentration levels fluctuated; they were first high then decreased on the 21st day of life, and then started to increase again until weaning. The high NEFA values at the beginning of life are considered to be related to the dam’s blood metabolic status, as it is well known that the blood NEFA value increases during the periparturient period. The increase in NEFA concentration level by the 45th day of life can be related to inadequate energy intake due to calf starter feed not being maximized yet. On the other hand, similar to the glucose value, blood NEFA values were lower in MPER25 and MPER50 than in other groups for the 21st and 42nd days of life (*p* < 0.0001). Moreover, they were the lowest in the control group on the 72nd day of life (Figure 1). Blood BHBA values linearly increased in all groups over time. However, as seen in glucose and NEFA values, blood BHBA levels differed in MPER25 and MPER50 and were lower than in other groups for the 21st and 42nd days of life (*p* < 0.0001) (Figure 1). The early decrease in glucose and increase in BHBA levels in MPER25 and MPER50 groups is considered as possibly being related to early rumen development. Linear increases were detected in both the root and leaf extract groups. NEFA and BHBA are the most valuable indicators for the energy metabolism of adult dairy cows [37]. Nevertheless, these parameters indicate different circumstances in newborn calves. NEFA is the first form of mobilized adipose tissue in blood and its increase points out the insufficient energy intake [38], whereas BHBA is also a metabolite of butyric acid in the liver [39]. An increased blood BHBA value in the first weeks of life is related to elevated butyric acid production due to the beginning of microbial growth in the rumen [40]. Therefore, an elevated blood BHBA concentration level without increased NEFA in newborn calves indicates the beginning of microbial activity and is related to early rumen development [41,42]. Administering different amounts of myrtle plant extract with milk to calves resulted in reduced blood glucose values in the MPEL-50 and MPER-50 groups. In a similar study [12], blood glucose concentration in laying hens, where 2.5%, 5% and 10% of myrtle plant extract were added to their drinking water, were found to be lower in all trial groups compared to the control group. Researchers attribute the hypoglycemic effect observed in the experimental groups to myricetin, the most abundant active ingredient in the myrtle plant extract. Myricetin, a hexahydroxyflavone, is significantly absorbed in the intestines of monogastric animals. Moreover, Ong and Khoo [43] reported that myricetin increased glucose transport in rat adipocytes without affecting insulin receptor function and Glucose Transporter Type 4 (GLUT-4) translocation [44]. They further noted that treatment of diabetic rats with myricetin led to a reduction in hyperglycemia. The significantly lower serum glucose level in the MPEL-50 and MPER-50 myrtle groups in this study may be explained by the hypoglycemic effect of myricetin. Similarly, Aljebory [11] and Saei et al. [28] reported that myrtle extract, given at a dose of 500 mg/kg, reduced blood glucose levels in rabbits and broilers, respectively. Various in vitro and in vivo studies have revealed that myricetin possesses the ability to alter the immune response or the functioning of the immune system by stimulating antibody formation. It was found to modulate LPS-stimulated activation of mouse bone marrow-derived dendritic cells (DCs) without inducing any toxic effects toward DCs at a concentration of 10 µg/mL. Dendritic cells are one of the immune cells constituting the mammalian immune system. Exposure to myricetin reduced the secretion of TNF-α, IL-6 and IL-12 in LPS-stimulated DCs [45]. In a study conducted to elucidate its mode of action, Kang et al. [46] suggested that myricetin primarily inhibited LPS-induced IL-12 production in mice. Furthermore, at concentrations of 5–100 μM, the compound inhibited mouse CD3 + T cell CD69 expression. Based on the above findings, it can be concluded that myricetin has the potential to modulate the immune system. 

In the presented study, the higher IgG values observed in the MPEL-50 and MPER-50 myrtle plant extract groups compared to the other groups suggest that this may be attributed to the dominant active ingredient in the myrtle plant extract, myricetin. Additionally, in the presented study, serum NEFA values of the MPEL-50 and MPER-50 myrtle plant extract groups were found to be lower than those of the other groups, while the serum BHBA values in the same groups were higher compared to the other groups. In a similar fattening study conducted on lambs, it was reported that peppermint oil added to the diets at a 3% level reduced the serum NEFA concentration compared to the control group in the initial period of the experiment but did not alter the BHBA levels [47]. Ster et al. [48] have emphasized that strategies preventing increases in blood NEFA levels during the transition period in dairy cows may limit postpartum immunosuppression. This explanation aligns with the observed decrease in NEFA values and increase in IgG values in the myrtle plant extract groups compared to the control group in our study.

In this study, no differences were observed between the groups regarding all blood count parameters except WBC (*p* < 0.0001), which is one of the blood physiology parameters (Table 6). On the other hand, hemoglobin and eosinophil values increased linearly in the MPER-25 and MPER-50 root extract groups compared to the control group, while the MCV values of the same groups decreased linearly. Similarly, in a study conducted by Taee et al. [29], where 0.5%, 1% and 1.5% myrtle powder were added to the basal diet of rainbow trout, WBC and hematocrit values increased in the blood of the experimental groups compared to the control group, while hemoglobin values did not change. In their study on broiler chickens, Bardzardi et al. [10] reported that myrtle extract reduced MCV and WBC values but did not change hemoglobin values. Salehifar et al. [49] also stated that myrtle extract (MEO), added to the basal diet in three different doses to broiler rations, increased the antibody titer, and that the 300 mg/kg MEO dose was the most effective (*p* < 0.05). In particular, broilers given MEO at 450 mg/Kg had lower levels of white blood cell count (WBC) and heterophile, heterophile/lymphocyte ratio, but higher lymphocyte and red blood cell counts (RBC) (*p* < 0.05), as shown by the study.

## 4. Conclusions

As a result, when extracts obtained from the leaves and root parts of the Myrtus (*Myrtus communis* L.) plant were given at dose levels of 25 and 50 mL/head × day for 72 days, with 60 days involving milk consumption, female Holstein calves were treated for diarrhea and pneumonia without experiencing any side effects. This treatment regimen not only reduced the incidence of these conditions but also had a positive impact on the increase in live weight and feed consumption. Consequently, the animals became pregnant at an earlier age through insemination, and the experimental groups required fewer inseminations. Additionally, upon evaluating blood physiology and biochemistry parameters, it was concluded that myrtle plant extract had a positive influence on metabolism and the immune system.

## Figures and Tables

**Table 1:** Ingredients and chemical composition of calf starter feed.

Ingredient	%
Corn grain	26
D.D.G.S. (Corn)	15
Soybean meal (CP 48%)	14.7
Corn Bran (%18P CP)	12.6
Wheat bran	12
Wheat grain	8
Sunflower meal (Dehulled, %36 CP)	7.9
Limestone	2.9
Salt	0.7
Premix *	0.2
Chemical Composition	
Dry Matter, %	88.19
Crude Protein, %	20.03
Metabolizable energy (KCal/kg)	2.622
Crude Fat, %	3.65
Crude Cellulose, %	7.39
Crude Ash, %	7.62
Ca, %	1.28
P, %	0.53
NDF, %	20.21
ADF, %	8.64

* 1 kg of Premix includes 15,000,000 IU of Vitamin A, 3,000,000 IU of Vitamin D_3_, 40,000 mg of Vitamin E, 100,000 mg of manganese, 100,000 mg of iron, 100,000 mg of zinc, 20,000 mg of copper, 1600 mg of iodine, 300 mg of cobalt, 300 mg of selenium.

**Table 2:** Chemical composition of the myrtle extract.

**Amino Acid Contents in the Myrtle Extract**																			
	Alanine	Arginine	Aspartic Acid	Cystine	Glutamic Acid	Glycine	Histidine	Isoleucine	Leucine	Lysine	Methionine	Proline	Serine	Threonine	Trytophan	Valine	Tyrosine	Phenylalanine	Norvaline
Leaf (mg/L)	8436.47	5484.26	10,193.61	1591.77	10,207.99	5759.73	3692.57	4567.86	7517.24	5971.68	900.21	32,886.81	11,543.61	9808.00	1386.40	7031.16	7265.74	4205.79	2982.68
Stem (mg/L)	6733.67	4268.64	9656.13	1366.17	9874.28	4572.21	3190.05	4260.04	5009.63	4329.44	769.37	4116.44	8799.76	7481.94	648.44	5547.04	5594.37	3607.42	252.44
**Active Ingredients of Myrtle Extract**																			
Leaf	Phthalic acid mono-2 ethylexyl ester	Gallic acid	Quinic Acid	Hydroxyhydroquinone/phloroglucin/Pyrogallol	Esculetin/Daphnetin														
Stem	Phthalic acid mono-2 ethylexyl ester	Hexadecylamine	Sodium tetradecyl sulphate	Nobuletin	3,5-di-tert-Butyl-4-hydroxybenzaldehyde														
Leaf plus Stem	Gallic acid	Vanillic acid	Catechin	Quercetin	Protocatechuic acid	Ellagic acid	Rosmarinic acid	Gentisic acid											
**Mineral Contents in Myrtle Extract**																			
Unit (mg/L)	Cadmium (Cd)	Lead (Pb)	Arsenic (As)	Mercury (Hg)	Magnesium (Mg)	Sodium (Na)	Iron (Fe)	Tin (Sn)	Aluminum (Al)										
Leaf	Not detected	0.008	0.002	0.002	36,000	335,000	1.46	1	1.26										
Stem	Not detected	0.007	0.002	0.003	25,000	328,000	2.04	1	1.24										

**Table 3:** Ingredients and chemical composition of heifer diet (TMR).

Feedstuffs	72 Days–6 Months	6–10 Months	6 Months—Pregnancy
	% DM	% DM	% DM
Corn silage. (Moderate Quality)	0	13.48	20.58
Alfalfa hay	35.6	30.82	21.23
Wheat straw	16.82	14.56	17.16
Corn. grain. fine ground	34.87	30.18	27.81
Canola meal (%34 CP)	4.89	4.23	6.69
Sunflower meal (Dehulled. %36 CP)	4.85	4.21	4.28
DCP	1.74	1.45	1.12
Salt	1.06	0.92	0.97
Premix *	0.17	0.15	0.16
Chemical Composition			
Dry Matter. %	89.91	70.26	62.87
Crude Protein. %	12.66	12.02	12.04
Metabolizable energy (KCal/kg)	2.19	2.21	2.22
Crude Fat. %	2.68	2.73	2.71
Crude Cellulose. %	21.48	22.51	22.49
Crude Ash. %	9.27	8.58	7.83
NDF. %	39.28	40.58	41.78
ADF. %	27.15	27.73	27.65

* 1 kg of Premix includes; 15,000,000 IU of Vitamin A, 3,000,000 IU of Vitamin D_3_, 40,000 mg of Vitamin E, 100,000 mg of manganese, 100,000 mg of iron, 100,000 mg of zinc, 20,000 mg of copper, 1600 mg of iode, 300 mg of cobalt, 300 mg of selenium.

**Table 4:** Effect of myrtle plant extract supplementation on performance of calves from 7 d to 72 d of age and subsequent age ^1^.

Item	Treatment						*p*-Values								
										Dose Response Leaves (%0–25–50) ^6^			Dose Response Root (%0–25–50) ^6^		
	Control	MPEL 25	MPEL 50	MPER 25	MPER 50	SEM ^5^	Treatment	Time	T × T ^≠^	C vs. T	Linear	Quadratic	C vs. T	Linear	Quadratic
BW ^2^*, kg	52.68	53.21	54.55	53.87	54.84	1.08	0.68	<0.0001	0.61	0.45	0.31	0.78	0.24	0.21	0.89
Feed Intake, g	9316.3 ^b^	9515.7 ^ab^	9830.7 ^a^	9611.8 ^ab^	9765.6 ^a^	90.0	0.002	<0.0001	0.99	0.01	0.001	0.48	0.01	0.004	0.80
FCR ^3^,g feed/g BW ^2^*	0.1647	0.1651	0.1663	0.1650	0.1649	0.003	0.98	<0.0001	0.82	0.73	0.67	0.97	0.94	0.92	0.98
BW ^2^** on day 300, kg	257.99 ^b^	266.71 ^ab^	268.41 ^a^	268.82 ^a^	268.62 ^a^	2.44	0.01	-	-	0.005	0.01	0.27	0.001	0.005	0.08
BW ^2^*** on day 410, kg	341.4	350.7	353.1	353.5	353.6	3.2	0.64	-	-	0.02	0.02	0.42	0.004	0.01	0.13
First AI ^4^, day	442.1	439.8	431.9	439.1	433.1	5.9	0.67	-	-	0.44	0.28	0.74	0.34	0.22	0.81
No. of AI/conception	1.6	1.5	1.2	1.3	1.2	0.2	0.39	-	-	0.31	0.16	0.68	0.11	0.11	0.64

^1^ Data are represented as least square means; Control: milk without supplement; MPEL 25: 25 mL/head × day Myrtus extract leaf supplemented milk; MPER 25: 25 mL/head × day Myrtus extract root supplemented milk; MPEL 50: 50 mL/head × day Myrtus extract leaf supplemented milk; MPER 50: 50 mL/head × day Myrtus extract root supplemented milk. ^2^* Body weight 72 days. ^2^** Body weight at 300 days of age. ^2^*** Body weight at 410 days of age. ^3^ Feed conversion ratio. ^4^ Artificial insemination. ^5^ Standard error of mean. ^6^ Coefficients of contrast for unequally spaced particle size levels were calculated using PROC IML of SAS. ^a,b^ Values with different superscripts in the same row are significantly different (*p* ≤ 0.05). ^≠^ T × T: Treatment × Time interaction; The treatment effects with time. Time-dependent effects of treatments.

**Table 5:** Effect of myrtle plant extract supplementation on serum biochemical parameters of calves from 7 d to 72 d of age ^1^.

Item	Treatment						*p*-Values								
										Dose Response Leaves (%0–25–50) ^6^			Dose Response Root (%0–25–50) ^6^		
	Control	MPEL 25	MPEL 50	MPER 25	MPER 50	SEM ^5^	Treatment	Time	T × T	C vs. T	Linear	Quadratic	C vs. T	Linear	Quadratic
Glucose, mg/dL	78.84 ^a^	78.22 ^ab^	76.13 ^ab^	79.25 ^a^	75.20 ^b^	0.84	0.01	<0.0001	<0.0001	0.14	0.05	0.54	0.15	0.005	0.02
NEFA ^2^, mmol/L	0.305 ^a^	0.299 ^a^	0.261 ^b^	0.309 ^a^	0.262 ^b^	0.005	<0.0001	<0.0001	<0.0001	0.002	<0.0001	0.02	0.01	<0.0001	0.0004
BHBA ^3^, mmol/L	0.360 ^c^	0.372 ^abc^	0.391 ^ab^	0.368 ^bc^	0.399 ^a^	0.007	0.0004	<0.0001	<0.0001	0.005	0.001	0.93	0.003	0.0001	0.23
Total protein, g/dL	6.52	6.62	6.73	6.74	6.69	0.07	0.15	0.01	0.93	0.08	0.04	0.89	0.02	0.09	0.11
BUN ^4^, mg/dL	15.35	15.26	16.01	15.86	16.23	0.26	0.56	<0.0001	0.42	0.36	0.07	0.18	0.03	0.02	0.85
IgG, mg/mL	6.02 ^b^	6.09 ^ab^	6.23 ^ab^	6.32 ^ab^	6.50 ^a^	0.13	0.05	<0.0001	0.13	0.36	0.21	0.71	0.01	0.004	0.70

^1^ Data are represented as least square means; Control: milk without supplement; MPEL 25: 25 mL/head × day Myrtus extract leaf supplemented milk; MPER 25: 25 mL/head × day Myrtus extract root supplemented milk; MPEL 50: 50 mL/head × day Myrtus extract leaf supplemented milk; MPER 50: 50 mL/head × day Myrtus extract root supplemented milk. ^2^ Non-esterified fatty acids. ^3^ Beta hydroxy butyric acid. ^4^ Blood urea nitrogen. ^5^ Standard error of mean. ^6^ Coefficients of contrast for unequally spaced particle size levels were calculated using PROC IML of SAS. ^a,b,c^ Values with different superscripts in the same row are significantly different (*p* ≤ 0.05). T × T: Treatment × Time interaction.

**Table 6:** Effect of myrtle plant extract supplementation on complete blood counts of calves from 7 d to 72 d of age ^1^.

Item	Treatment						*p*-Values								
										Dose Response Leaves (%0–25–50) ^9^			Dose Response Root (%0–25–50) ^9^		
	Control	MPEL 25	MPEL 50	MPER 25	MPER 50	SEM ^8^	Treatment	Time	T × T	C vs. T	Linear	Quadratic	C vs. T	Linear	Quadratic
RBC ^2^, 10^6^/dL	7.15	7.00	7.03	7.09	7.31	0.12	0.41	<0.0001	0.43	0.43	0.54	0.61	0.71	0.34	0.35
Hematocrit, %	25.68	25.62	25.79	25.94	25.83	0.12	0.32	<0.0001	0.64	0.86	0.52	0.45	0.19	0.42	0.22
Hemoglobin, g/dL	9.19	9.31	9.30	9.23	9.38	0.08	0.48	<0.0001	0.68	0.21	0.31	0.47	0.30	0.14	0.60
MCV ^3^, fL	37.92	37.95	37.69	37.77	37.63	0.14	0.43	<0.0001	0.90	0.55	0.25	0.43	0.18	0.13	0.99
MCH ^4^, pg	12.45	12.44	12.60	12.57	12.47	0.08	0.53	<0.0001	0.93	0.46	0.19	0.42	0.55	0.93	0.30
MCHC ^5^, g/dL	31.01	31.15	31.13	31.17	31.08	0.17	0.96	0.04	0.10	0.53	0.63	0.66	0.56	0.73	0.57
RDW ^6^, %	41.17	41.27	41.20	41.03	41.24	0.15	0.83	0.03	0.75	0.76	0.92	0.65	0.87	0.73	0.35
Platelet, 10^9^/L	420.52	420.45	423.68	419.70	419.60	2.82	0.84	0.86	0.38	0.68	0.46	0.65	0.80	0.82	0.91
WBC ^7^, 10^9^/L	7.45 ^b^	7.74 ^ab^	7.92 ^a^	7.54 ^b^	7.96 ^a^	0.07	<0.0001	0.005	0.47	<0.0001	<0.0001	0.51	0.003	<0.0001	0.07
Neutrophil, %	49.58	49.49	49.37	49.60	49.06	0.24	0.53	<0.0001	0.97	0.66	0.57	0.93	0.40	0.13	0.31
Lymphocytes, %	39.38	38.93	38.89	39.26	39.09	0.29	0.70	<0.0001	0.20	0.24	0.28	0.61	0.59	0.48	0.88
Monocytes, %	9.37	9.18	9.59	9.50	9.54	0.17	0.38	<0.0001	0.63	0.93	0.37	0.18	0.44	0.45	0.81
Eosinophil, %	0.89	0.89	1.07	1.08	1.11	0.08	0.08	0.19	0.21	0.70	0.23	0.19	0.04	0.07	0.30
Basophils, %	0.262	0.258	0.268	0.265	0.273	0.024	0.78	0.04	0.42	0.97	0.66	0.40	0.52	0.59	0.72

^1^ Data are represented as least square means; Control: milk without supplement; MPEL 25: 25 mL/head × day Myrtus extract leaf supplemented milk; MPER 25: 25 mL/head × day Myrtus extract root supplemented milk; MPEL 50: 50 mL/head × day Myrtus extract leaf supplemented milk; MPER 50: 50 mL/head × day Myrtus extract root supplemented milk. ^2^ Red blood cell count. ^3^ Mean corpuscular volume. ^4^ Mean corpuscular hemoglobin. ^5^ Mean corpuscular hemoglobin concentration. ^6^ Red cell distribution width. ^7^ White blood cell count. ^8^ Standard error of mean. ^9^ Coefficients of contrast for unequally spaced particle size levels were calculated using PROC IML of SAS. ^a,b^ Values with different superscripts in the same row are significantly different (*p* ≤ 0.05). T × T: Treatment × Time interaction.

## Data Availability

All the data obtained during this study are contained within this article. No new data, other than presented in the current article, are created.
